# A novel method for subgroup discovery in precision medicine based on topological data analysis

**DOI:** 10.1186/s12911-025-02852-9

**Published:** 2025-03-19

**Authors:** Ciara F. Loughrey, Sarah Maguire, Paweł Dłotko, Lu Bai, Nick Orr, Anna Jurek-Loughrey

**Affiliations:** 1 School of Electronics, Electrical Engineering and Computer Science, Queen’s University Belfast, Belfast, UK; 2 Patrick G Johnston Centre for Cancer Research, Queen’s University Belfast, Belfast, UK; 3 Dioscuri Centre in Topological Data Analysis, Mathematical Institute, Polish Academy of Sciences, Warsaw, Poland

**Keywords:** Topological data analysis, Hotspot detection, Breast cancer, Patient stratification, Subgroup discovery

## Abstract

**Background:**

The Mapper algorithm is a data mining topological tool that can help us to obtain higher level understanding of disease by visualising the structure of patient data as a similarity graph. It has been successfully applied for exploratory analysis of cancer data in the past, delivering several significant subgroup discoveries. Using the Mapper algorithm in practice requires setting up multiple parameters. The graph then needs to be manually analysed according to a research question at hand. It has been highlighted in the literature that Mapper’s parameters have significant impact on the output graph shape and there is no established way to select their optimal values. Hence while using the Mapper algorithm, different parameter values and consequently different output graphs need to be studied. This prevents routine application of the Mapper algorithm in real world settings.

**Methods:**

We propose a new algorithm for subgroup discovery within the Mapper graph. We refer to the task as hotspot detection as it is designed to identify homogenous and geometrically compact subsets of patients, which are distinct with respect to their clinical or molecular profiles (e.g. survival). Furthermore, we propose to include the existence of a hotspot as a criterion while searching the parameter space, addressing one of the key limitations of the Mapper algorithm (i.e. parameter selection).

**Results:**

Two experiments were performed to demonstrate the efficacy of the algorithm, including an artificial hotspot in the Two Circles dataset and a real world case study of subgroup discovery in oestrogen receptor-positive breast cancer. Our hotspot detection algorithm successfully identified graphs containing homogenous communities of nodes within the Two Circles dataset. When applied to gene expression data of ER+ breast cancer patients, appropriate parameters were identified to generate a Mapper graph revealing a hotspot of ER+ patients with poor prognosis and characteristic patterns of gene expression. This was subsequently confirmed in an independent breast cancer dataset.

**Conclusions:**

Our proposed method can be effectively applied for subgroup discovery with pathology data. It allows us to find optimal parameters of the Mapper algorithm, bridging the gap between its potential and the translational research.

**Supplementary Information:**

The online version contains supplementary material available at 10.1186/s12911-025-02852-9.

## Introduction

Patient subgroup discovery using pathology data is a critical area of research aimed at identifying meaningful subgroups of patients with similar disease characteristics, outcomes, or treatment responses. This problem is particularly relevant in precision medicine, where the goal is to tailor treatments based on individual variability in disease biology. Pathology data, including histological images, gene expression profiles, proteomics, and clinical information, provide a rich source of information for uncovering hidden patterns and heterogeneity within patient populations. The challenge lies in the high dimensionality and complexity of these datasets, coupled with the need to ensure that identified subgroups are clinically interpretable and biologically meaningful. Common approaches to patient subgroup discovery include unsupervised learning techniques, such as clustering algorithms. Methods like k-means, hierarchical clustering, and Gaussian Mixture Models are widely used for partitioning data into subgroups based on similarity. Additionally, dimensionality reduction techniques like Principal Component Analysis (PCA), t-SNE, and UMAP are often employed to preprocess data and visualize the subgroup structure in lower-dimensional space.Topological methods have recently emerged as a reliable and interpretable framework for extracting information from high-dimensional data, leading to the field of computer science and mathematics called Topological Data Analysis (TDA) [1]. TDA provides tools rooted in computational geometry and topology for summarising the shape of multidimensional data. It builds on concepts in algebraic topology to robustly summarise key topological features such as loops, clusters, or flares that persist within a dataset.

The Mapper algorithm is a TDA tool used to uncover and visualize the shape and structure of high-dimensional data. By incorporating dimensionality reduction and local clustering, it creates a visual representation of the data’s underlying topology by building a network graph [1]. This graph represents key structures of the dataset clearly while preserving local relationships between samples. The dimensionality reduction is performed across a guiding lens function. This lens function can be based on mathematical properties of the data (e.g. sample means), classical dimensionality reduction techniques (e.g. PCA) or focused research questions (e.g. patient survival). When applied with pathology data, the Mapper algorithm offers a unique approach to subgroup discovery. As opposed to commonly applied clustering techniques (e.g. agglomerative clustering), which group data points into disjoint or overlapping clusters based on similarity metrics, Mapper represents data as a graph where nodes correspond to clusters of data points in overlapping subsets. This graph emphasizes relationships and connectivity between subgroups, providing a more nuanced view of the data structure. It excels at discovering complex topological features in the data, such as loops (e.g., recurrent states), branches (e.g., disease progression pathways), and voids (e.g., absence of certain phenotypes), which may be oversimplified or missed in the standard clustering or dimensionality reduction process.

TDA methods have become increasingly popular within biological applications [2–4] as they can detect interesting topological structures (e.g. loops or flares) which traditional methods such as clustering techniques may struggle to find.

The Mapper algorithm was used to obtain new insights from data in diverse healthcare applications including organisational mapping of brain activity from MRI data [5], identification of three core Type 2 diabetes subgroups from electronic health records [6], and the characterisation of recurrent patterns in genomic data from viral evolution events [7]. It has also successfully identified subgroups of interest in breast cancer [8–10]. A new subgroup with poor survival and high ER expression was discovered from gene expression data that was refractory to detection using standard clustering analysis [8]. The traditional 50-gene signature classification method, PAM50, which identifies five molecular intrinsic subtypes of breast cancer, was refined to deconstruct these into seven subtypes [9]. By applying Mapper to transcriptional data transformed to reveal the extent that diseased tissue deviates from healthy tissue, termed “Disease-Specific Genomic Analysis” (DSGA) [11], a cohort of oestrogen receptor-positive (ER+) patients was found to contain a novel subgroup with extremely good survival rates [10].

While Mapper has proved to be an effective data mining tool for biomedical data, it is currently not applicable in diagnostic settings as manual selection of its parameters is required to generate a relevant graph that highlights important aspects of biology within the dataset. Multiple parameters must be defined by the user when setting up the Mapper algorithm and these decisions can strongly affect the final output, resulting in variable groupings of patients [8, 12]. Additionally, once a Mapper graph is constructed, the output graph must be manually inspected to assess whether any meaningful and relevant phenomena are in fact revealed. The research trying to address Mapper parameter selection is very limited. There has been some work carried out to improve the selection of parameters and establish stability in the output of Mapper analysis. In [13] the authors proposed to use a stability measure to select Mapper cover parameters. The stability was measure as a pairwise distance between Mapper graphs constructed across different partitions of the dataset. It was assumed that lower average distances signifies greater stability. However, choosing a single parameter combination according to this strategy remained challenging due to multiple potentially "stable" representations. Furthermore, stability doesn’t ensure useful data representation as too few nodes can omit important details, and many isolated nodes yield a stable yet useless configuration.

In their work in [14], the authors used Fuzzy Silhouette Score to identify optimal parameter values. The ten highest-scoring graphs were combined using an ensemble method adapted from [15]. Using the cluster-similarity metric from [15], a correlation matrix was constructed, with each element representing sample composition correlation of node pairs. Following this, the correlation matrix was converted to a distance matrix, and hierarchical clustering was applied to the nodes to construct the final graph. It is worth noticing that Silhouette scores may be inconsistent indicators of graph quality, as the metric favours convex clusters over non-convex.

In [16], the authors proposed F-Mapper as an alternative to the standard Mapper algorithm. F-Mapper uses Fuzzy C-Means clustering [17] to define the coverage over the lens with irregular intervals. As a soft clustering method, Fuzzy C-Means assigns a value between 0 and 1 to each sample for each of the *c* clusters. F-Mapper uses a threshold parameter where each sample is assigned to any cluster with a value greater than the threshold. The algorithm then clusters on the pullback of the overlapping sets defined by Fuzzy C-Means clusters. The authors fine-tuned the parameters to topologically match the output of the standard Mapper on known datasets and found that F-Mapper outperformed the standard Mapper in terms of Silhouette score [18]. However, the lens, *c* parameter, and threshold parameter in F-Mapper are user-chosen.

The authors of [19] addressed the Mapper’s parameter selection challenged by incorporating the concept of ensemble learning in the Mapper graph construction process. A pool of Mapper graphs was initially constructed, which were then ranked based on their stability and prevalence. The top graphs were then ensembled in the final output graph.

Apart from parameter selection challenge, there also isn’t much work proposing automated methods for detecting subgroups within Mapper graphs. A study by [20] focused on subgroup identification using Mapper graphs and proposed a pipeline for ranking clusters in the graph. A pool of graphs was constructed using different parameter values and the most representative and statistically significant topological features (i.e. clusters) were selected. The cluster were then ranked according to the separation regarding to the chosen outcome of interest. The top clusters were analysed as potential patients subgroups.

In this work we propose a novel solution, which addresses the two aforementioned problems simultaneously. Firstly, we propose a new algorithm for detection of hotspots within Mapper graph, which can be used as a tool for patient subgroup discovery. Secondly, to address the parameter selection problem within the Mapper graph, we propose to use the existence of pre-defined phenomena within the graph of patients (i.e. existence of a hotspot) as a selection criterion for the Mapper parameters. We believe that this type of automation could help to bridge the gap between the potential of the Mapper algorithm and the translational research. We present two experiments demonstrating the effectiveness of our proposed technique. As a simple example study, an artificial hotspot present in a toy Two Circles dataset is identified. Next, we apply our methodology in a ER+ breast cancer case study, decoding heterogeneous groups of patients according to differing survival outcomes.

## Preliminaries

### The Mapper algorithm

**Fig. 1:**
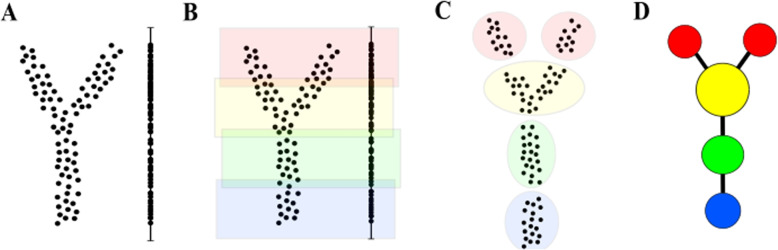
Simple workflow of the Mapper algorithm. **A** Data points are projected via a lens function. **B** The projected values are divided into overlapping intervals. **C** Clustering is carried out within each interval. **D** A graph is built on the clustering

Here we introduce the Mapper algorithm originally proposed by Singh et al. (2007) [1]. Figure 1 visualises the main steps of the algorithm using toy data as an example. The Mapper takes as input a set of data points $X=\{x_1, \dots , x_k\}, x_i \in \mathbb {R}^n$ with a notion of distance described between two data points.

In the first step, all data points are projected into low dimensional space (usually one or two dimensions) via the application of a **lens function** ($f : X \rightarrow \mathbb {R}^l$, $l \in \{1,2\}$). The lens function is typically chosen to highlight data qualities relevant to the research question, such as a particular feature or combination of features. In the case of $l=1$, as per Fig. 1A, the dataset *X* is projected to a set of real-number values.

In the second step, the range of the projected values (*f*(*X*)) is divided into (or covered with) **t** intervals that overlap on **p** percentage of their length (Fig. 1B). For instance, if $f(X) = [0,1]$, **t**=4 and **p**=20%, the coverage will consist of the following four intervals of length $\frac{5}{17}$: $\left[0,\frac{5}{17}\right], \left[\frac{4}{17},\frac{9}{17}\right], \left[\frac{8}{17},\frac{13}{17}\right]$ and $\left[\frac{12}{17},1\right]$ .

In the third step, for every interval *I* from the coverage of *f*(*X*) a clustering algorithm is run on the data points from *X* having their projected values in *I*. The clustering is performed in the original space $R^n$. Mathematically we would say that the clustering is performed on the preimage $f^{-1}(I) = \{ x \in X | f(x) \in I \}$. As an output, for each interval *I* we obtain a collection of clusters $C^I_1,\ldots ,C^I_m$ (Fig. 1C). Finally, the graph is constructed where each cluster corresponds to a single vertex. Two clusters $C^I_i$ and $C^J_k$ are joined by an edge if they contain any overlapping data points. Mathematically, $C^I_i$ and $C^J_k$ are connected if $C^I_i \cap C^J_k \ne \emptyset$. As the final output, we obtain a graph as per Fig. 1D, which could be then coloured based on the value of a selected attribute (e.g. survival). More precisely, the value of each vertex of the mapper graph will correspond to an average value of the attribute for the points in the corresponding cluster.

One may consider the Mapper algorithm as a combination of dimensionality reduction and clustering. Unlike clustering, Mapper does not make any assumption regarding the shape of data (i.e. data is dividable into clusters). Its aim is to visualise the shape of the data via a particular lens and hence it can not only detect clusters but also other different topological structures of the data like loops or flares. In comparison to existing dimensionality reduction/visualisation methods, Mapper also has a winning aspect. The clustering within the Mapper algorithm is happening in the original space, which means that the graph highlights substructures from the original space. This is not the case with other dimensionality reduction methods that suffer from precision loss (well separated data points in the original space may be projected close to each other).

**Fig. 2:**
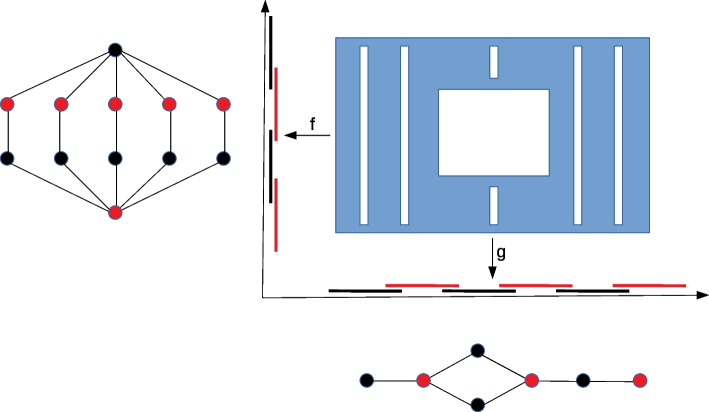
Example of two Mapper graph constructions. Consider the blue set of dense data points. We consider two possibilities of the lens functions: *f* and *g*. The appropriate pullbacks and clustering provide the Mapper graphs on the left (for the function *f*) and in the bottom (for the function *g*)

As discussed earlier, the key challenge with applying the Mapper graph is the selection of its parameter values. The example in the Fig. 2 highlights that different choices of lens functions, for example, may provide different Mapper graphs. It is a natural phenomenon, as lens functions determine which details of the image are being neglected.

### What are hotspots?

**Fig. 3:**
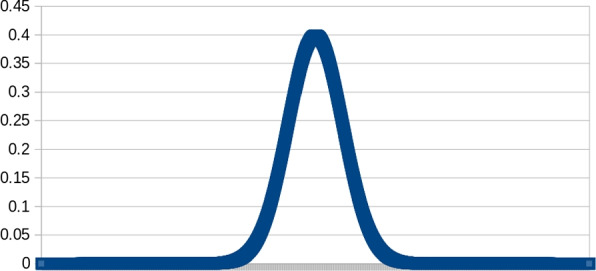
A normal distribution. The region of the domain close to the centre of the plot represents a hotspot of considerably higher values

In this section we will build an intuition and explain what a hotspot is. For a given function $f : D \rightarrow \mathbb {R}$, a hotspot is a small, connected and compact sub-region $D'$ of the domain *D* on which *f* has considerably lower, or considerably higher values than in the neighbourhood of $D'$. As an example of this simple case, consider the domain $D = [-10,10]$ and the normal distribution with an average 0 and standard deviation 1 as presented in Fig. 3. A region of the domain close to the mean value of the distribution, for instance $D' = [-1,1]$, is a hotspot, as the values therein are considerably higher than in a further neighbourhood $D'$. Note that it is difficult to precisely define the range of hotspot. In this study we will make it dependant on certain parameters.

This leads us to consider how a hotspot may be in a continuous setting; we need to locate a point $x \in D$ and two neighbourhoods $N_s^x$ and $N_l^x$ where $N_s^x \subset N_l^x$ such that the averaged value of *f* in $N_s^x$ is considerably larger or smaller than the averaged value of *f* on $N_l^x$. This intuition works well provided we can effectively locate neighbourhoods of points in the domain *D*. In our case, the space *D* is modelled by a Mapper graph. It is important to note that two data points that are considered to be close to each other on the Mapper graph, may be far away from each other in Euclidean sense (i.e. for two data points that are close according to the Mapper graph, the Euclidean distance between them may be high).

As demonstrated by aforementioned literature [6, 8–10], hotspot detection is a critical task in precision medicine, focusing on identifying regions or subsets of data that exhibit significantly higher activity or frequency of a specific event, such as overall survival, genetic mutations, or adverse drug reactions. By identifying regions or populations with elevated risks or specific characteristics, hotspot detection could facilitate early diagnosis, tailored treatments, efficient resource allocation, and effective public health interventions.

## Methods

The purpose of our algorithm is to search for unusual small regions (hotspots) within a given dataset. We define a hotspot as a relatively small region of the considered space with the values of the function of interest being considerably different than in the neighbourhood of the region. The hotspot searching algorithm is applied on a constructed Mapper graph. In this context, we define hotspots as structurally connected and homogenous community of nodes that present heterogeneous behaviour with respect to a characteristic of interest (e.g. survival) in the context of the surrounding neighbourhood nodes. During Mapper parameter selection, different lenses considered by the user can produce alternate perspectives on the underlying shape of the input data and this results in multiple different graph outputs, as shown in Fig. 2. Our algorithm can explore the space of possible Mapper graphs that can be obtained from the input data at hand to find a combination of parameters (if exists), which reveals the existence of a hotspot of interest. The algorithm allows the user to inspect diverse perspectives of the graph space for a hotspot by sampling different lenses, and subsequently the two parameters covering the lens (the number of intervals *t* and the overlap *p*). The clustering settings (i.e. clustering algorithm and its parameters) are not considered in the searching process but could also be incorporated. The algorithm runs in three stages, which are (1) Construction of the Mapper graph, (2) Community detection and (3) Community classification. We describe each of the steps in the following subsections. The pseudo-code of the hotspot detection stages (2 & 3) is presented in Algorithm 1.

**Figure:**
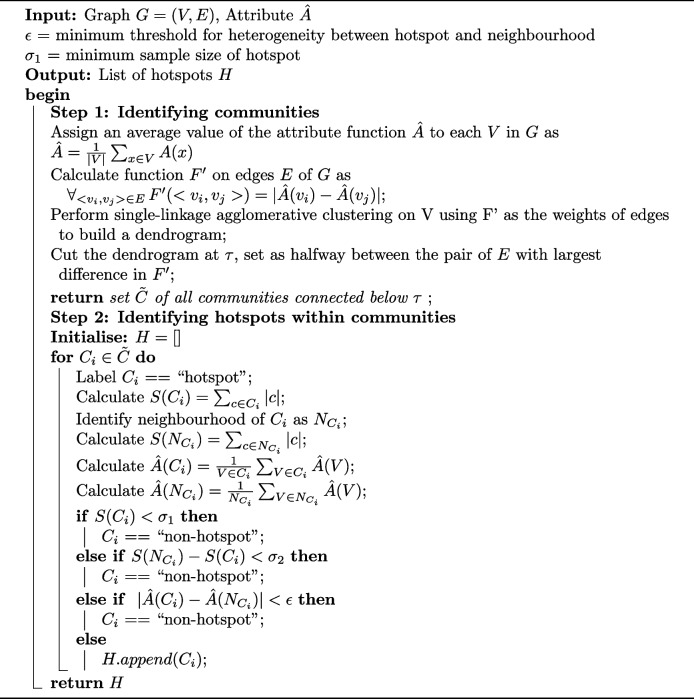
**Algorithm 1** Hotspot detection algorithm

### Construction of the Mapper graph

We propose to construct a Mapper graph by automatically sampling the parameter space and using the hotspot detection algorithm to evaluate graphs for the presence of anomalous nodes. We consider different ranges of values for the number of intervals and the overlap parameters. Those could be predefined by the user. In our implementation we consider the most intuitive and commonly used values (“Breast cancer dataset” section). For the lens function we propose to consider a random linear combination of all possible features with at most $50\%$ of coefficients being non-zero. Mathematically, for a given set of $n-$dimensional data points $X \subset \mathbb {R}^n$ where $x=(x_1,\ldots ,x_n) \in X$ a lens function is calculated as per Eq. 1.

1 $$\begin{aligned} f(x) = \alpha _1 x_1 + \alpha _2 x_2 + \ldots + \alpha _n x_n \end{aligned}$$

where values of coefficients $\alpha _i$ are either set to zero or are randomly uniformly sampled from an interval $[-2.5,2.5]$. Note that those lens functions are Lipschitz continuous and the presented process may be viewed as feature selection. To allow for significant feature reduction, we only consider a subset of the number of features for each lens function. The randomly selected subset of features is different for every lens function, by defining a zero-value weight for the rejected features and a unique randomly sampled non-zero weight for the selected elements. Therefore within a single search, a lens function is generated with new weights and hence a new subset of non-zero features. Pre-selection of informative variables can also be carried out on the input point cloud prior to the construction of the lens function. For a combination of a function from the *lens* space and the values of *t* and *p*, a Mapper graph *G* is constructed. Following construction of the graph, the hotspot detection algorithm is executed in two following steps.

### Community detection

In the first step, the algorithm searches for non-intersecting connected components of *G* that are homogeneous with respect to an attribute of interest $\hat{A}$ (e.g. survival), considered as communities of vertices. Each community will further be classified as hotspot or non-hotspot. Note that there are cases when those regions are not uniquely defined. Therefore, we chose any subset that satisfies the internal homogeneity criteria. The process runs as follows; Given *G*, vertices are assigned an average value of an attribute function $\hat{A}$ (e.g. survival time) - the average is calculated given vertex *v* as per Eq. 2.

2 $$\begin{aligned} \hat{A}(v) = \frac{1}{|v|}\sum \limits _{x\in v}A(x) \end{aligned}$$

Following this, we define a function on edges, $F'$, capturing gradient of $\hat{A}$ over the edges of the graph. The value of $F'$ for an edge is calculated as the absolute difference in $\hat{A}$ between its two connecting vertices, as shown in Eq. 3.

3 $$\begin{aligned} \forall _{{<}v_{i},v_{j}{>}\in E}\, F'(<v_{i},v_{j}>)=|\hat{A}(v_{i})-\hat{A}(v_{j})| \end{aligned}$$

Function $F'$ can be consider as a distance function between two vertices. We assume that distance from a vertex to itself is equals to zero ($\forall _{v\in V}\, F'(v)=0$). In the next step single linkage clustering is performed on vertices from *G* using $F'$ as the distance function. Consequently, two vertices connected by an edge with similar $\hat{A}$ values will merge quickly, as the value of the edge will be small. Given two vertices joined by an edge with very different values of $\hat{A}$, the edge joining them will appear late in the merging process. The cut-off point in the dendrogram, determining the parameter $\tau$ is calculated as the largest difference between the histogram edge value rankings (i.e. the largest distance between horizontal branches in the dendrogram). This cut-off selects clusters of components containing homogenous vertices connected over longer timespans in the dendrograms. The output of community detection is a set of communities $\tilde{C}=\{C_1, \dots , C_n\}$ of interconnected vertices within *G* which are homogenous in consideration to $\hat{A}$.

### Community classification

In the final step, each community is checked against a set of conditions to assess whether it constitutes a hotspot or not. Community classification ensures hotspot size and neighbourhood heterogeneity meets user-defined thresholds. The size *S* of the community group corresponds to the number of samples contained within its vertices. The hotspot size must be larger than a predefined minimum sample size $\sigma _1$. We assume that if a community $C_i$ is very small then it should be considered as an outlier rather than a hotspot. At the same time for a community to be a hotspot it should be proportionally smaller than its neighbourhood. We propose that the difference between the size of a hotspot $C_i$ and its neighbourhood $N_{C_i}$ is larger that the median absolute deviation of all identified communities ($S(C_1), \dots , S(C_n)$). Simultaneously, the difference in $\hat{A}$ between the hotspot and the neighbourhood (all other connected vertices within the graph) must be greater than a pre-defined parameter $\epsilon$. This ensures hotspots only occur within the extreme ranges of the attribute function and provide flexibility in the definition of an anomalous subgroup. We assume that $\epsilon$ and $\sigma _1$ should be set by the user as they strongly depend on the domain and $F'$. The community classification step follows for each $C_i \in \tilde{C}$:

1. Consider the neighbourhood $N_{C_i}$ as the collection of remaining vertices in $\tilde{C}$ which are not found within $C_i$
2. Calculate size $S(C_i)$ and $S(N_{C_i})$ as the number of samples *n* contained in the vertices of $C_i$ and $N_{C_i}$, respectively
3. Calculate $\hat{A}(C_i)$ and $\hat{A}(N_{C_i})$ as the mean value of $\hat{A}$ across all vertices within $C_i$ and $N_{C_i}$ respectively.
4. Calculate $\sigma _2$ as median absolute deviation of $S(C_1), \dots , S(C_n)$
5. If $S(C_i) < \sigma _1$ or $S(N_{C_i}) - S(C_i)< \sigma _2$ then classify $C_i$ as a non-hotspot. This restricts the search to hotspots that lie in the lower range of community sizes and ensures the hotspot does not cover a large proportion of the graph.
6. If $|\hat{A}(C_i) - \hat{A}(N_{C_i})|> \epsilon$ then $C_i$ is considered as a hotspot. Otherwise $C_i$ is classified as a non-hotspot.

The complete hotspot detection algorithm is summarised in Algorithm 1. There are two parameters that need to be specified by the user. The first determines the minimum size of a hotspot, and the second determines the minimum difference in the attribute of interest between the hotspot and its neighborhood. It is important to note that these parameters are domain- and problem-specific. Their values are typically intuitive for the user, who is likely a domain expert (e.g., a clinician). In contrast, selecting a lens function and the two cover parameters of the Mapper algorithm is a challenging task, even for individuals with technical knowledge and understanding of the algorithm, let alone users from the medical domain. Therefore, replacing the selection of Mapper parameters with these two domain-specific parameters makes the algorithm significantly more clinically applicable.

### Data and implementation details

#### Two circles dataset

**Fig. 4:**
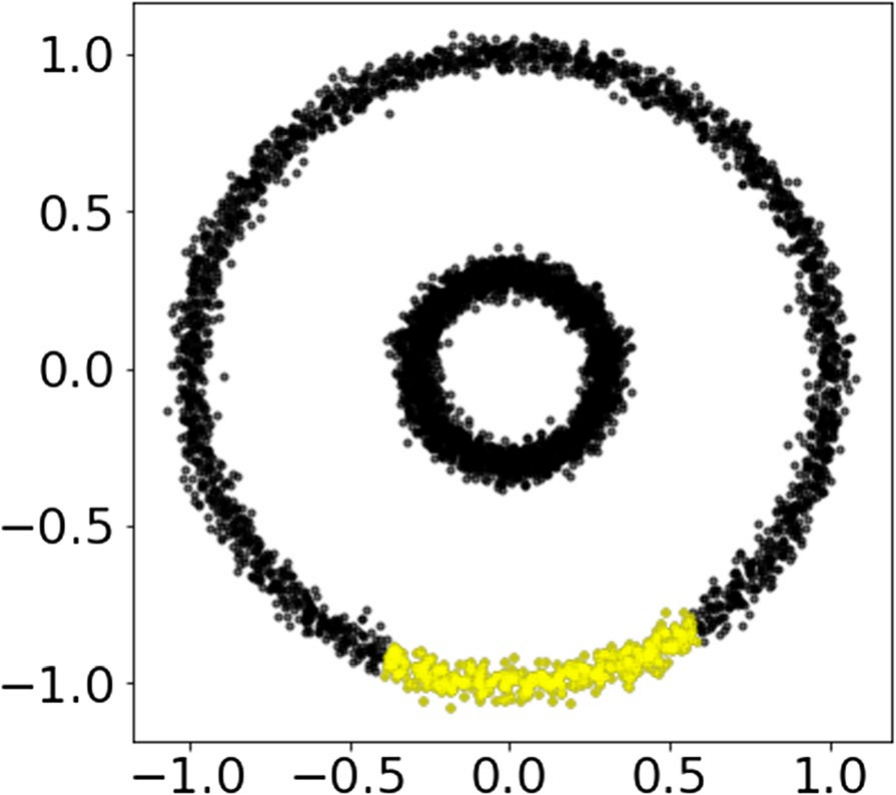
Toy dataset. Two disconnected circles with an artificially predefined hotspot highlighted in yellow

In our first experiment, we applied our hotspot detection algorithm to a simple toy dataset consisting of 5000 samples and two features, which forms the shape of two concentric circles (Fig. 4). We artificially labelled a portion of samples as Class 1, representing 8.1% of the samples ($n=405$) in the outer ring. The remaining 91.9% samples are classified as Class 2.

We searched for a hotspot by sampling from the parameter space. Lens functions were generated as described in “Construction of the Mapper graph” section. Both features were assigned non-zero weights during lens generation as we did not perform feature selection due to the small size of the dataset. Intervals values across the range of [5,10,15,20,25] and an overlap percentage of [40%, 60%, 80%] were considered. The agglomerative hierarchical clustering algorithm with 6 clusters and ward’s linkage was used to build the graph [21]. The hotspot detection algorithm parameters were set to $\epsilon = 0.5$ and $\sigma _1 = 30$. We set the attribute function as Class 1 labels to search for a hotspot containing a higher proportion of Class 1 samples.

### Breast cancer dataset

For the second experiment we used two breast cancer tumour gene expression datasets that had detailed clinicopathological annotation, namely the Molecular Taxonomy of Breast Cancer International Consortium (METABRIC) and The Cancer Genome Atlas (TCGA) breast cancer dataset [22, 23]. We divided the datasets into a discovery (METABRIC) and a validation (TCGA) set. To pre-process both datasets for analysis with Mapper we performed DSGA-transformation, a method which subsets a selection of genes highlighting the deviation of disease from a healthy phenotype and previously shown to improve the distinction of subgroups in breast cancer when combined with the Mapper algorithm [10, 11]. A dataset of gene expression from healthy breast tissue obtained from the Genotype-Tissue Expression (GTEx) data repository was used as the healthy state model during DSGA processing [24]. Detailed descriptions of data accession and preprocessing steps are provided in S5 File. DSGA-transformation with gene thresholding produced a gene expression matrix of 575 genes.

As per the steps of the proposed algorithm, different combinations of Mapper parameters were used to construct Mapper graphs which were interrogated for the presence of a hotspot. For each combination of parameters, if a hotspot was not present, the graph was rejected and new parameters values were generated. Lenses were generated from a linear combination of features specifying at most 50% of the $\alpha _i$ weights as non-zero, as described in “Construction of the Mapper graph” section, to allow us to modify the weighted effect of each gene across a search while simultaneously acting as a feature reduction technique. The interval range was set between 10 and 30 by increases of 2 and the overlap range between 10% and 45%, over increases of 5%. Pairwise combinations of intervals and overlap culminated in 88 combinations. For a generated lens, graphs were built for every parameter pair with the objective of identifying a hotspot. Euclidean distance was used to calculate the distance between two samples. The HDBSCAN [25] clustering algorithm was implemented with default parameters. The attribute function was defined as the occurrence of relapse before 10 years, colouring the nodes of the graph. In addition to the proposed lens function, we also evaluated a number of dimensionality reduction methods commonly applied as lens for the Mapper algorithm: Principle Component Analysis [26] (extracts the most significant axes (principal components) that capture the largest variance in the data) and UMAP [27] (reduces high-dimensional data into 2D or 3D embeddings that preserve local or global similarity), t-SNE [28] (reduces dimensions by preserving the local relationships between data points) and Isomap [29] (reduces dimensions by preserving the global geometric structure of data), applying one of the reduced dimensions as the lens.

When a graph was generated the hotspot detection algorithm searched for a hotspot presenting high occurrence of relapse before 10 years in relation to the neighbourhood. To define our attribute function, samples were labelled as either ‘1: Relapse occurs before 10 years’ or ‘0: No relapse before 10 years’. The minimum sample size for a hotspot was set at 30 patients and $\epsilon$ was defined as 0.1, searching for hotspots with high occurrence of relapse before 10 years. An $\epsilon$ value of 0.1 corresponds to a 10% difference in the proportion of samples in the community cluster experiencing a relapse event in relation to the neighbourhood. Log-rank test were performed for any hotspots, thresholding significance at $P < 0.05$, comparing the 10-year relapse-free survival (RFS) of hotspot patients against the neighbourhood patients. If more than one hotspot were present for a generated lens, hotspots were ranked by log-rank *p*-value and the hotspot group with the strongest difference in survival outcome was selected for further analysis. It should be noted that instead of selecting just one hotspot based on the log-rank *p*-value, the hotspots can be analysed collectively instead. The Kaplan-Meier method was used to compare RFS for the successful hotspot against the corresponding neighbourhood group. When a hotspot was identified as significant for a lens function, the search ended and no more lens fuctions were considered.

Following a successful discovery of a hotspot, we searched for the presence of a similar hotspot group in an independent breast cancer dataset to validate our methodology. We performed an automated search for hotspots on the validation dataset, using the same lens function found in the successful METABRIC hotspot search. Note that because the data came from different sources we allowed for different values of the other two parameters (i.e. intervals number and overlap) to be considered for the validation dataset. As the values ranges may differ between the two datasets it may not be possible to obtain the same hotspot for the exact same values of those parameters. The hotspot detection parameters were kept the same. For all interval and overlap combinations containing a hotspot, the RFS of hotspot groups and their neighbourhoods were compared by log-rank tests and ranked by *p*-value. The hotspot group with the strongest difference in survival outcome was selected as the TCGA hotspot group and Kaplan-Meier analysis was performed comparing the confirmed TCGA hotspot and its neighbourhood group. The similarity of the validation hotspot group to the discovery hotspot group was investigated. The centroid of the METABRIC hotspot class was calculated according to the genes identified in the lens function. The distance of TCGA patients to this centroid was measured using Canberra distance. Canberra distance was chosen as it is sensitive to values close to zero, hence it is well suited for DSGA-transformed z-score values of gene expression. The validation Mapper was coloured by the distance of each patient to the centroid, confirming the validation hotspot group represents patients with the highest similarity in gene expression to the discovery hotspot group.

## Results

The hotspot detection algorithm can be used in conjunction with the Mapper algorithm to identify a hotspot of interest in a graph and simultaneously perform automatic parameter selection. To demonstrate this we initially apply our methodology to an artificial exemplar dataset. Following this, we implement the hotspot detection pipeline using a real world case study of subgroup discovery in cancer research.

### Two concentric circles

**Fig. 5:**
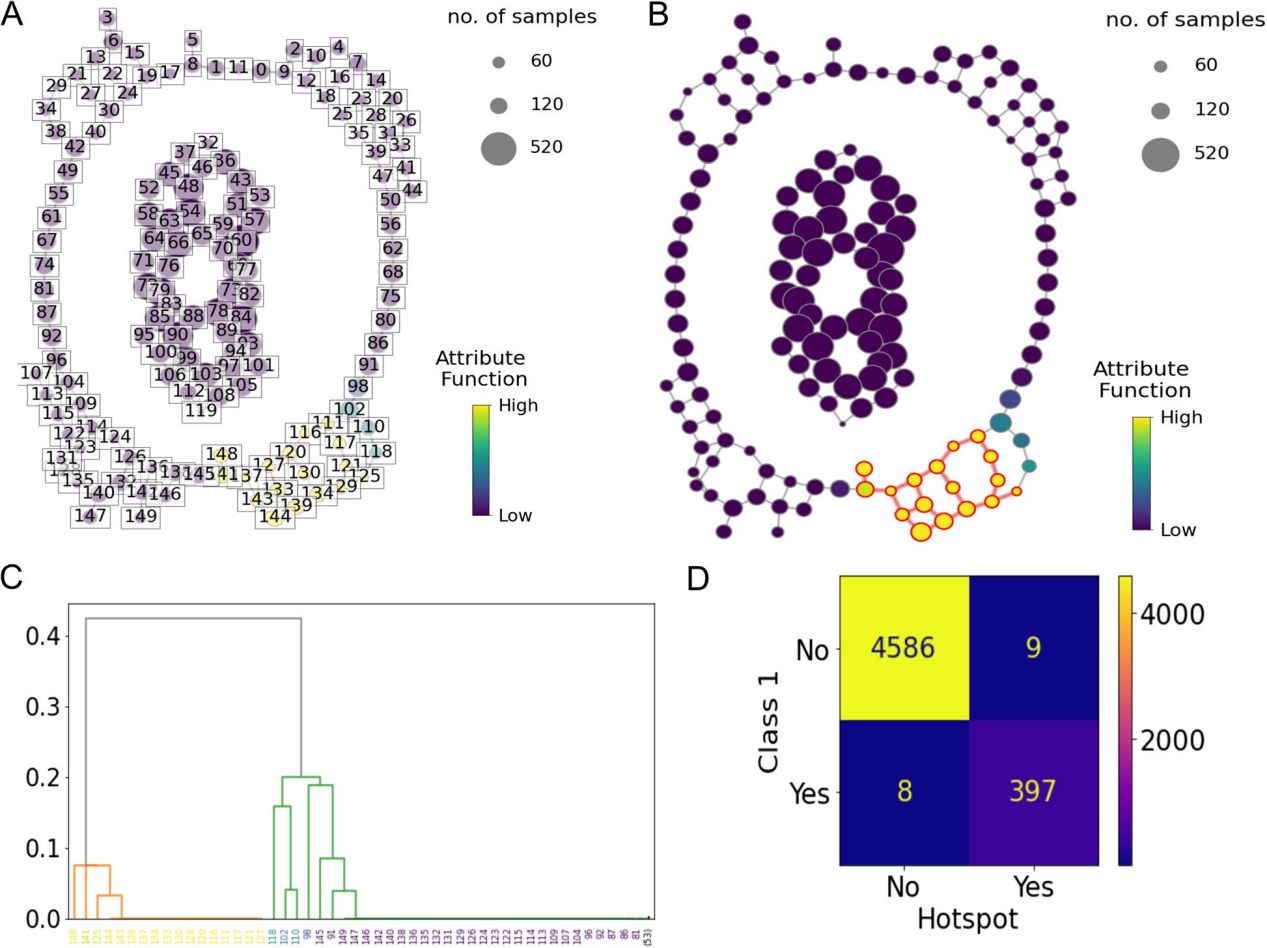
Visualisation of the proposed hotspot detection algorithm for a toy dataset. **A** The Mapper algorithm outputs two graph components corresponding to each circle of the graph. Nodes are coloured by the attribute and labelled from 0 to 149. Node size reflects the number of samples in a node. **B** The identified hotspot is highlighted in red in the Mapper graph. Nodes are unlabelled. **C** Dendrogram representing the output of the single linkage algorithm performed on the nodes of the Mapper component corresponding to the outer circle from Fig. 4 in the community detection phase. Two community clusters are present and node labels are coloured by average attribute value on the x-axis. The dendrogram is truncated to improve visualisation due to the large number of nodes with zero-value edge weights in the graph. **D** The confusion matrix comparing the samples contained in the hotspot to the samples originally labelled as Class 1

A successful lens function was identified, weighting the x-axis by 0.747 and the y-axis by −0.827. Five covers of the lens function were found to contain a hotspot and these five combinations of interval and overlap values were ranked by the proportion of Class 1 samples present. The final parameter combinations were 25 intervals with 80% overlap (Fig. 5A). The final hotspot is contained in nodes [129, 130, 133, 134, 137, 140, 141, 111, 143, 145, 115, 116, 148, 121, 123, 124, 126], highlighted in Fig. 5B. It can be noted from the dendrogram in Fig. 5C that two community clusters were detected (coloured as orange and green) in the outer circle of the graph. No hotspots were identified in the Mapper graph component built on the inner circle of the dataset. The hotspot contains 98% of the original Class 1 samples, achieving an f1-score of 0.979 (Fig. 5D).

### Investigating relapse in oestrogen-positive breast cancer

We applied our algorithm firstly to a discovery cohort of ER+ patients, searching the parameter space for a lens function that can construct a Mapper graph containing a hotspot (representing a cluster of patients with poor prognosis). Following this, we show that the identified lens function can build a Mapper graph containing a similar hotspot subset of patients in an independent validation cohort. The molecular and clinical profile of the hotspot subset present in both datasets is investigated.

#### Discovery analysis in METABRIC cohort

**Fig. 6:**
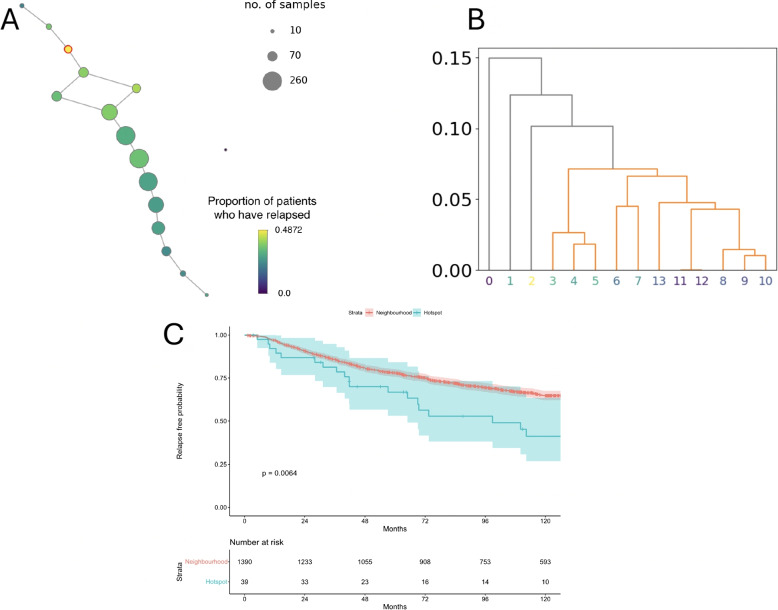
Representation of the ER+ METABRIC cohort through the Mapper graph. **A** The Mapper graph built using parameters identified from the hotspot detection algorithm run on METABRIC data. Each labelled node represents a group of patients with similar gene expression. The node size represents the number of patients covered by it as indicated in the top right legend. The bottom right legend describes the attribute function colouring the graph. In this instance, this is the occurrence of relapse before 10-years. The node identified as a hotspot is highlighted in red. **B** The dendrogram obtained from the cluster identification step of the hotspot detection algorithm. Nodes labels are coloured by attribute function. Node 2 is identified as a single cluster within the Mapper graph. **C** Kaplan-Meier curve differentiating RFS outcome in months between the hotspot group and the neighbourhood group of patients

The algorithm was applied on the METABRIC dataset of 1429 ER+ breast cancer patients. Our search identified a lens function with 222 non-zero weighted genes which led to a Mapper graph containing a hotspot. These genes and their corresponding weights are listed in S1 Table. The Mapper graph is visualised in Fig. 6A. Parameters for the final Mapper graph were selected based on the top-ranking hotspot according to survival outcome, set at 24 intervals with 10% overlap ($P = 0.006$) (Fig. 6C). We were not able to identify a hotspot when any of the four dimensionality reduction methods (i.e. PCA, UMAP, i-SNE, Isomap) were applied as the lens function. This highlights the importance of the lens searching strategy proposed in the paper.

The graph split into four clusters according to survival outcome (Fig. 6B). A hotspot was present in node 2, shown in Fig. 6A Mapper graph (Table 1). This node represented a cluster containing 39 patients (2.73% of the cohort) and the survival probability of these individuals living relapse-free until 10 years is 41% compared to a survival probability of 65% for the rest of the cohort. The clinicopathological characteristics of the METABRIC hotspot patients are provided in S2 Table.

**Table 1:** Hotspot detection results

	METABRIC	TCGA
Node label	2	1
Hotspot sample size	39	30
Neighbourhood sample size	1390	760
Hotspot survival probability	41%	54%
Neighbourhood survival probability	65%	59%

#### Validation in the TCGA cohort

We evaluated whether the hotspot identified in the METABRIC dataset could be independently replicated by searching for the presence of a similar hotspot in the TCGA validation cohort of 790 ER+ breast cancer patients. We performed the parameter search across the interval and overlap range using the successful lens function with 222 non-zero weighted genes identified for the METABRIC dataset. The hotspot detection algorithm parameters were not changed.

**Fig. 7:**
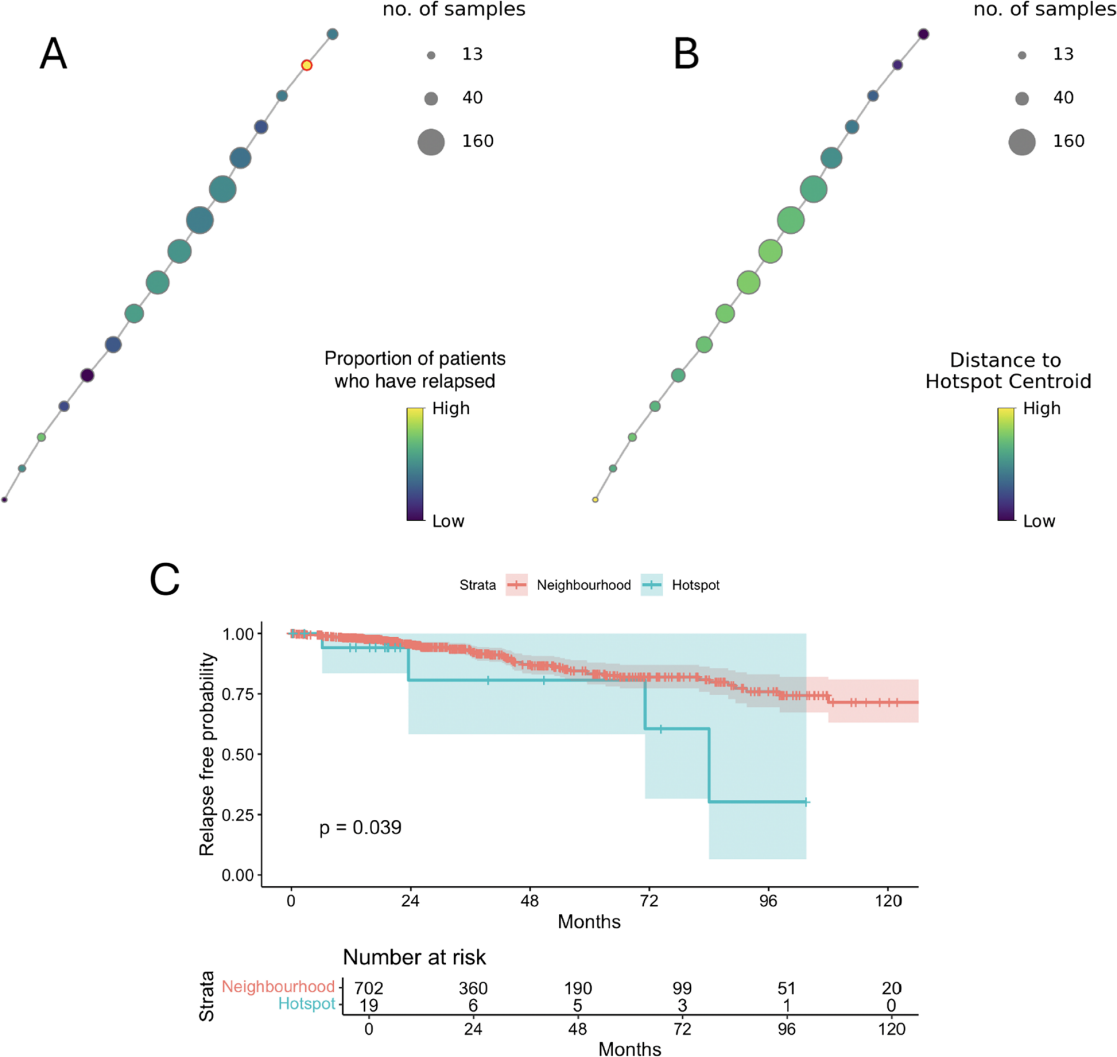
Representation of the ER+ TCGA cohort through the Mapper graph. **A** The Mapper graph built on the TCGA data using the lens function of 222 genes identified from the hotspot detection algorithm results for the METABRIC data. Each labelled node represents a group of patients with similar gene expression. The node size represent the number of patients covered, indicated in the top right legend. The bottom right legend describes the attribute function colouring the graph, which is the occurrence of relapse before 10-years. Hotspot detection identified highlighted in red as a hotspot. **B** Mapper graph coloured by the distance of TCGA patients to the METABRIC hotspot centroid according to the 222 lens function genes. **C** Kaplan-Meier curve differentiating OS survival outcome in months between the hotspot group and the remaining ER+ cohort

Number of interval and percentage of overlap parameters were selected according to the top-ranking hotspot by survival outcome. The final Mapper graph for the TCGA cohort was set at 30 intervals with 30% overlap ($P = 0.04$) (Fig. 7D). After running hotspot detection, one node was confirmed as the successful hotspot node (Fig. 7B). This node represented 19 individuals or 2.71% of the total ER+ patients. The graph was also coloured by the distance of the TCGA cohort to the METABRIC hotspot centroid across the 222 genes identified by the lens function (Table 1 B). The hotspot node is confirmed as the cluster of patients with the highest similarity to the METABRIC hotspot group (Fig. 7B).

#### Molecular and clinical profile of the hotspot group

Breast cancer tumours can be classified into intrinsic subtypes, including luminal A, luminal B, HER2, basal, claudin-low, and normal-like based on gene expression. These subtypes are associated with differences in patient survival [30, 31]. In the METABRIC dataset, sixteen tumours in the hotspot were classified as *Luminal A* and ten tumours as *Luminal B*. The remaining thirteen tumours were *Claudin-low* (six patients), *Her2* (three patients), and two patients each to the subtypes *Basal* and *Normal-like*. There was no evidence on an association between tumour intrinsic subtype and the hotspot subgroup (chi-square $P=0.29$). Similarly in the TCGA dataset, no association was found between tumour intrinsic subtype and the hotspot subgroup (chi-square $P=0.07$).

**Fig. 8:**
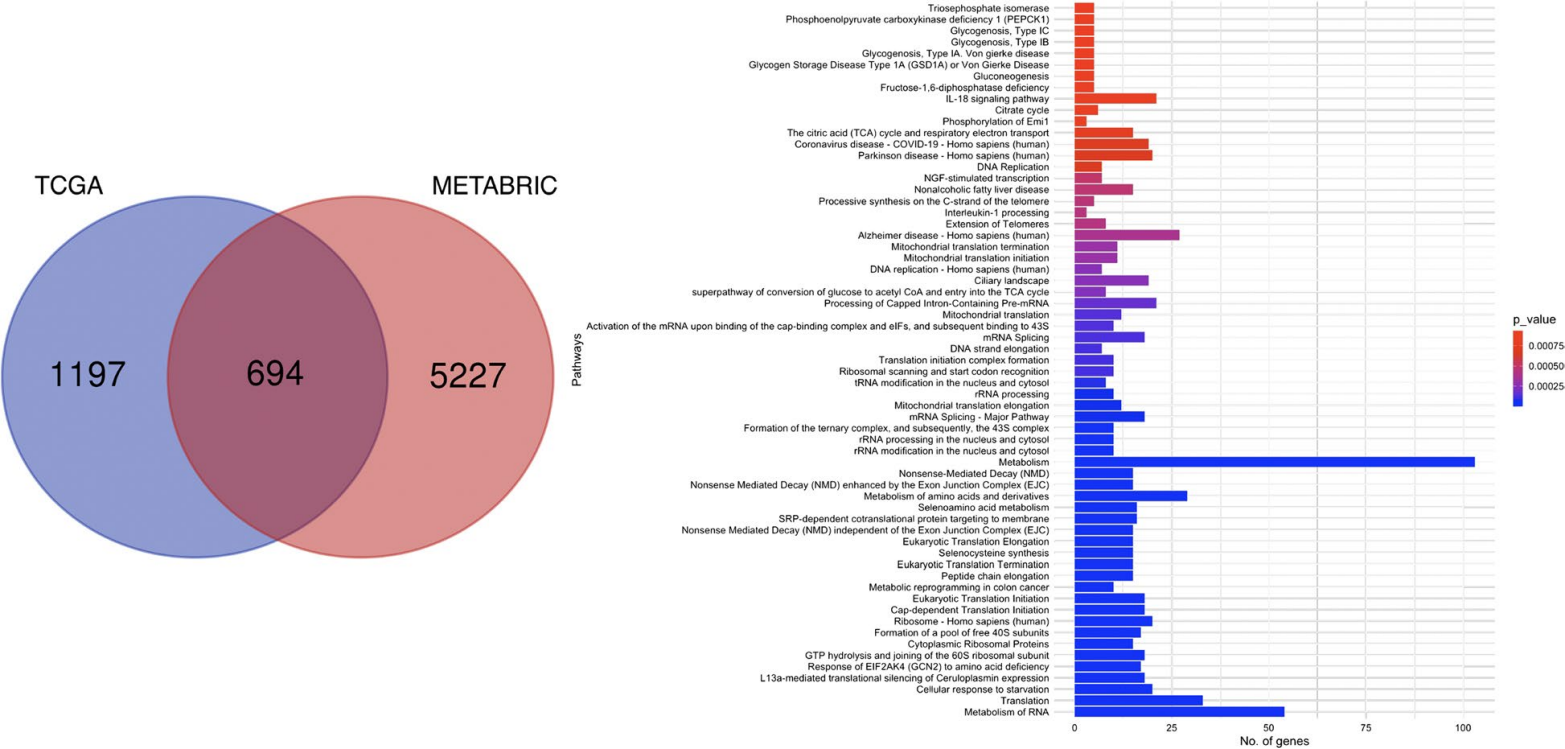
Pathway analysis results for the hotspot group. **A** Comparison of differentially expressed genes shared between the METABRIC and TCGA hotspot group. **B** Biological pathways over-represented in the shared gene set of 694 differentially expressed genes. The y-axis represents the identified pathways and the x-axis corresponds to the number of genes from the gene set involved in that pathway. Bars are coloured by the significance value

We investigated the underlying biology of the hotspot. There were 5,227 unique differentially expressed genes in the METABRIC dataset and 1,197 significant genes in the TCGA dataset, of which 694 were present in both (Fig. 8A). Pathway enrichment was performed for the shared enriched gene set of 694 genes. Pathway enrichment results for the shared gene list can be found in S3 Table. 63 pathways were significant and 483 genes (70.1%) from the input list are present in at least one pathway and the results are visualised in Fig. 8B.

## Discussion

Our hotspot detection algorithm successfully identified graphs containing homogenous communities of nodes within the Two Circles dataset. When applied to gene expression data of ER+ breast cancer patients, a lens function was constructed and appropriate parameters were identified to generate a Mapper graph containing a hotspot. This hotspot highlighted a group of ER+ patients with poor prognosis and characteristic patterns of gene expression, subsequently confirmed in an independent breast cancer dataset. We compared the composition of the hotspot group from existing classifications available for breast cancer samples. The identified patient group was not represented by any of the well-known PAM50 subtypes, which are important indicators of prognostic status according to a 50-gene signature assay [32]. Our hotspot detection algorithm identified the presence of a subgroup with high occurrence of relapse representing 2.73%- 3.79% of ER+ tumours, which cannot be assigned to existing PAM50 subtypes. This indicates that the hotspot detection algorithm carries out refined stratification of a complex disease. The discovery hotspot persists over several interval and overlap parameter selections for the identified lens function. While these parameters can introduce deformations to the graph clustering, the reoccurrence of the hotspot group over several parameter perturbations reduces the likelihood of the hotspot being a random artefact.

The validity of our approach is further confirmed by replicating results in the validation dataset. For this analysis, a Mapper graph was built on TCGA data using the identical lens function identified from the discovery algorithm search (Fig. 7A). We separated a group of patients with the highest similarity in gene expression to the METABRIC hotspot group (Fig. 7B) and poor prognostic outcome (Fig. 7C). This confirms the relevance of the hotspot group in wider breast cancer datasets and the importance of the identified gene signature for differentiation of the poor prognostic group from the ER+ cohort. The molecular profile of the hotspot groups was additionally investigated to identify why this group of patients was clustered independently from the other ER+ patients. Differential expression analysis identified 694 shared genes differentiating the METABRIC and TCGA hotspot group. This gene list was significantly enriched in 63 pathways, described in S3 Table, many of which are linked to tumour progression and development in breast cancer and novel areas of therapeutic treatment within the literature.

Selecting appropriate parameters of the Mapper’s algorithm that will reveal a subgroup is a time-consuming process for current users, particularly researchers in bioinformatics without extensive knowledge in TDA. The introduction of an evaluation method to assess the quality of the graph according to how it stratifies a cohort into subgroups can improve the application of Mapper for subgroup discovery in precision medicine. Carr et al., 2021 demonstrate how homogenous subgroups of patients can be identified as node clusters composing interesting topological features, such as flares [20]. In this paper and further demonstrated by Iniesta et. al, 2022 [33], Mapper graphs for different parameter combinations are ranked by the impurity of clusters according to a variable of interest to compare homogeneity of clusters. Our hotspot detection algorithm similarly evaluates parameter combinations for the Mapper graph and discriminates graphs which contain homogenous clusters of patients representing a subgroup of interest. However, our method differs substantially as we define the concept of a subgroup as a smaller sub-region of the graph that is heterogenous relative to its neighbourhood. Our concept of a cluster in the graph is not necessarily distinguished by the structure of the graph, but by the relationship between the cluster and neighbourhood of the cluster characterized by the attribute of interest.

## Conclusions and future work

We have presented an automated process of hotspot detection using topological data analysis which can stratify genomic data to highlight biologically important groups of patients. Our method augments the TDA technique Mapper by automatically detecting clusters of anomalous patients in the graph in consideration to an attribute of interest. The lens function, number of intervals, and percentage of overlap parameters influence the mapping of the original dataset to the graph representation and can generate numerous graph outputs. The hotspot detection algorithm supports automatic selection of values for these parameters by evaluating graphs for hotspots. The algorithm allows the user to investigate a specific research question for subgroup discovery. We demonstrated this application by focusing our search on groups of individuals experiencing high prevalence of survival events to find a group of ER+ breast cancer patients with poor prognosis. This approach improved the applicability of the Mapper method and made it easier to be used in clinical settings by reducing the requirements for an intensive manual search of parameters. Simultaneously, the method filters a high dimensional collection of genes to a small gene signature containing biologically relevant information. Our new algorithm provides a refined approach to discriminate sub-types within heterogenous diseases while simultaneously simplifying the application of the Mapper algorithm in exploratory data analysis for bioinformatics.

As an extension of this study, we recognise the importance of evaluating the generalisability of our proposed algorithm across a broader range of cancer types and datasets. While this paper focuses on demonstrating the novelty of our method and its utility through the discovery and comprehensive validation of a novel hotspot in breast cancer, future work will involve applying the algorithm to additional cancers, leveraging datasets such as TCGA, which provides multi-omics and clinical data across a wide variety of cancer types. This will allow us to assess whether the identified subgroups exhibit significant prognostic differences across different cancers and explore their alignment with established clinical and molecular subtypes. By undertaking such analyses, we aim to validate the broader applicability of the algorithm and further demonstrate its potential as a robust tool for cancer subtyping and precision medicine.

We would further like to expand the hotspot detection method to support the integration of multiple datatypes, as the collection of multi-omics datasets is ever increasing in bioinformatics. Collating data from several sources can achieve more accurate cancer prognosis prediction by providing a systems-level overview of the different levels of biological relationships driving disease [34]. The impact of the described pathways needs to be investigated further to confirm a causal relationship between the survival outcome and disrupted pathway events.

## Supplementary information

Supplementary Material 1: S1 Table. Selected gene features and weights for the identified lens function

Supplementary Material 2: S2 Table. Clinicopathological characteristics of patients in the METABRIC dataset

Supplementary Material 3: S3 Table. Clinicopathological characteristics of patients in the TCGA dataset

Supplementary Material 4: S4 Table. Summary of pathway analysis results for the shared gene list

Supplementary Material 5: S5 Data access and pre-processing

## Data Availability

There are no primary data in the paper and data accession is described in S5 File. Code is available on a GitHub repository at https://github.com/annajl/hotspotdetection.
