# Supplementary material for: A novel method for subgroup discovery in precision medicine based on topological data analysis
Source: BMC Med Inform Decis Mak. 2025 Mar 19;25:139. doi: 10.1186/s12911-025-02852-9 (PMC11921513; doi:10.1186/s12911-025-02852-9)
Supplement: Supplementary file 1 — Supplementary Material 1: S1 Table. Selected gene features and weights for the identified lens function [file 12911_2025_2852_MOESM1_ESM.pdf]

Genes	Weights
CTTN	0.128094
ATP6V0A4	0.85213
BSN	-0.37108
SPAG6	0.459627
DEFB1	-0.46106
COL2A1	0.274234
PNOC	0.340818
PIGX	-0.24376
KRT5	1.418006
HOGA1	-1.92994
ZNF148	-0.66649
EVI5	-0.88779
FCRLB	0.200019
IL18	-0.15499
UGT2B10	-0.11588
GNG4	0.680155
NEU4	0.942522
SORCS1	0.83553
CHRD	0.413562
RADIL	0.173939
RBM24	0.378144
ADH1B	0.069658
IGDCC3	0.192516
TNFRSF17	0.066951
BANK1	1.975569
ALG8	-0.7927
PIGR	-0.27881
GSK3B	-0.6613
SHROOM1	0.826841
FGF10	-1.15249
MSH3	0.006515
PVALB	-0.20758
ELAVL1	0.148371
VSIG2	-0.36307
YAF2	-0.16378
PI15	-0.91393
NKX3-2	0.836369
TERF1	-1.63586
MRPL57	0.116557
TUBA3C	-0.2845
SP5	-0.48943
GPR1	-0.52139
JSRP1	-0.74751
IL17RD	0.041349
LAMP3	1.69666
SLC51A	-0.2765
KCNH1	-0.89804
SUZ12	0.15259
SLC5A1	1.35149

HSPB6	0.280873
NKX3-1	-0.83225
RAB11FIP1	-0.80014
LEP	0.717487
POTEB3	1.565695
PLVAP	0.885974
TRPV6	-0.14085
PHGR1	1.847137
ANO3	-0.52222
MRPS16	0.07394
TAF13	-0.76703
DISP2	0.302736
CLEC3A	0.966722
LRRC31	0.765461
NPY1R	0.119796
VCX3A	-1.35262
MKX	0.369164
SLC1A1	0.651856
TYW3	0.137085
CHGB	0.154663
AGTR1	0.199443
MCOLN3	0.013233
ALPP	-0.35905
TMEM74B	2.230749
LRP1B	0.790093
EIF4EBP1	0.064805
SFRP1	-0.1663
RIMS4	0.448702
IRF8	-0.55815
INSM1	-0.98546
ECM1	-0.547
KCNF1	0.358399
KYNU	-0.40394
CYP4F12	-0.93326
KCNC2	0.262078
KCNE4	0.600221
TECTA	-0.73457
SSTR2	-0.40815
ROBO2	-1.89693
ZNF706	0.178186
CHST1	-0.87854
MSMB	0.149379
RASSF10	0.179572
CLNS1A	-0.25392
CCBE1	-0.50344
CEP19	0.355531
BEX1	0.148015
TEKT1	-0.68304
EDN3	-0.16333
CBLN2	-0.36084

NAPSA	0.158388
UPK1A	0.868874
PBX1	1.203624
GALNT5	0.183741
ZNF562	0.870022
NOL10	-1.82283
PHAX	0.453311
ITPK1-AS1	0.506749
PCDHA3	-0.54432
ROPN1B	-0.80852
FAM155A	-0.47173
NUCKS1	-0.64306
UGT2B11	-0.55172
NFS1	0.05422
PHF21B	0.826424
GNA14	0.468838
SSX2	-0.09126
SERPINA1	-0.98611
ALG1L	1.818112
CETN3	-0.54298
ASIC1	0.291583
FFAR2	-0.21683
MCMDC2	-0.82931
S100A7	-0.18671
ROPN1	1.091005
TUBAL3	0.404755
PLPP5	-0.92246
CXCL17	-0.64183
TRH	-0.17728
NFYB	0.439076
ABCC12	0.937723
PNPT1	-0.7126
HOXB-AS3	0.605912
FAM3B	0.26514
TEX14	0.276948
PRR11	-0.67068
VPREB3	-0.91791
PRKCQ-AS1	-1.09466
FCGBP	-0.16887
TFPI2	0.250515
ABHD12B	-0.07034
ST6GALNA	0.003728
CREB1	0.102539
PTGR2	-0.12682
HOXB6	-1.83882
UBXN2A	0.794508
NARS2	0.842696
TNFAIP8	0.223182
BLOC1S6	0.079024
LINC00960	-0.60484

LILRB1	-0.53553
SPINK8	-0.92181
TNNI3	-1.30566
TRIM36	0.038535
C4orf3	0.115322
NOVA1	0.142849
AAMDC	0.004644
CLDN20	0.99702
ZNF14	-0.58576
CD38	0.947964
RDH16	-0.2194
ATP8B3	-0.96309
SCNN1B	0.06484
SERPINI1	-0.79089
SEMA3E	-0.11877
TMEM267	-0.20788
CLCA2	-0.41667
CD19	-0.42377
DAPL1	1.215155
KLRD1	-0.9978
CA11	-0.15915
GNB4	0.466116
POU2AF1	-0.12792
ARL16	0.589055
STAC2	-0.26531
KCNH2	0.544189
CYP26A1	-1.25703
ERLIN2	-0.66913
GOLGA8CP	-0.75066
KRT14	0.591331
TMEM151A	-0.29392
OSR1	1.513991
EMX1	1.966241
C1QTNF3	-0.17487
SLITRK6	0.221928
TPSG1	1.492833
LIME1	0.70245
SLC22A18A	-0.40382
NAPB	0.728674
C4orf19	0.482737
VTRNA1-3	0.231404
SKA2	-0.37534
OSR2	0.859488
FLT3	0.50898
SOX2	-0.67232
IRAK3	0.978383
RNF19A	0.027457
SLC26A3	0.537615
TBC1D32	0.997449
TSHZ2	-0.39686

VIPR2	-0.58134
TUBB2B	-0.23943
HOXA5	0.424052
FAM189A1	0.24618
GUCY1A2	-0.99272
CARTPT	1.168973
RIMS2	-0.83861
MED15P9	0.323108
TDG	-0.67695
SYCE1	-0.25574
PKN2	-0.74749
DMC1	-0.98742
S100A9	0.044799
CHRNA5	0.702918
ANKRD46	-0.29392
ASPHD1	0.836753
ADAMDEC	-0.1972
ASH2L	0.073339
N4BP2	-0.77905
ZYG11B	0.441595
PLCB4	0.92019
FRAS1	0.612056
NFASC	0.245993

S1 Table. Selected gene features and weights for the identified lens function.
