# Supplementary material for: A novel method for subgroup discovery in precision medicine based on topological data analysis
Source: BMC Med Inform Decis Mak. 2025 Mar 19;25:139. doi: 10.1186/s12911-025-02852-9 (PMC11921513; doi:10.1186/s12911-025-02852-9)
Supplement: Supplementary file 2 — Supplementary Material 2: S2 Table. Clinicopathological characteristics of patients in the METABRIC dataset [file 12911_2025_2852_MOESM2_ESM.pdf]

CLINICAL VARIABLE	ALL CASES (N = 1429)	HOTSPOT (N = 39)
**Age at Diagnosis**		
Median (IQR)	63.92 (54.81 - 72.17)	66.76 (54.17 - 74.95)
**Neoplasm Histologic Grade**		
1	154	5
2	665	11
3	553	21
NA	57	2
**Tumour Other Histologic Subtype**		
Ductal / NST	1051	33
Lobular	125	1
Medullary	7	0
Mixed	185	4
Mucinous	19	1
Other	14	0
Tubular / cribriform	18	0
NA	10	0
**Lymph Nodes Examined Positive**		
>0 Nodes	655	18
0 Nodes	774	21
**Tumour Size**		
Median (IQR)	22 (17 - 30)	23 (17.95 - 30)
**Tumour Stage**		
1	379	7
2	598	14
3	70	1
4	9	0
NA	373	17

S2 Table. Clinicopathological characteristics of patients in the METABRIC dataset.
