# Supplementary material for: A novel method for subgroup discovery in precision medicine based on topological data analysis
Source: BMC Med Inform Decis Mak. 2025 Mar 19;25:139. doi: 10.1186/s12911-025-02852-9 (PMC11921513; doi:10.1186/s12911-025-02852-9)
Supplement: Supplementary file 3 — Supplementary Material 3: S3 Table. Clinicopathological characteristics of patients in the TCGA dataset [file 12911_2025_2852_MOESM3_ESM.pdf]

CLINICAL VARIABLE
**Diagnosis Age**
Median (IQR)
**American Joint Committee on Cancer Tumor Stage Code**
T1
T1b
T1c
T2
T2a
T3
T3a
T4
T4b
TX
**Positive Finding Lymph Node Hematoxylin and Eosin Staining Microscopy Count**
>0 Nodes
0 Nodes
NA

S3 Table. Clinicopathological characteristics of patients in the TCGA dataset.

ALL CASES (N = 790)	HOTSPOT (N = 30)
59 (49 - 69)	63.5 (51.25 - 71)
29	0
14	1
168	3
441	20
1	0
109	5
1	0
4	0
21	1
2	0
363	17
301	8
126	5
