# Supplementary material for: A novel method for subgroup discovery in precision medicine based on topological data analysis
Source: BMC Med Inform Decis Mak. 2025 Mar 19;25:139. doi: 10.1186/s12911-025-02852-9 (PMC11921513; doi:10.1186/s12911-025-02852-9)
Supplement: Supplementary file 4 — Supplementary Material 4: S4 Table. Summary of pathway analysis results for the shared gene list [file 12911_2025_2852_MOESM4_ESM.pdf]

Pathway	Database	No. of genes
ADORA2B mediated anti-inflammatory cytokines production	Reactome	4
Renin secretion - Homo sapiens (human)	KEGG	3
Endochondral Ossification	Wikipathways	3
Endochondral Ossification with Skeletal Dysplasias	Wikipathways	3
Long-term depression - Homo sapiens (human)	KEGG	3
Phosphodiesterases in neuronal function	Wikipathways	3
MicroRNAs in cardiomyocyte hypertrophy	Wikipathways	4
ion channels and their functional role in vascular endothelium	BioCarta	3
Salivary secretion - Homo sapiens (human)	KEGG	4
actions of nitric oxide in the heart	BioCarta	3
G alpha (s) signalling events	Reactome	5
Platelet homeostasis	Reactome	4
Endothelin Pathways	Wikipathways	3
Platelet activation - Homo sapiens (human)	KEGG	5
Calcium signaling pathway - Homo sapiens (human)	KEGG	7
Signal Transduction	Reactome	28
Muscle contraction	Reactome	7
cGMP-PKG signaling pathway - Homo sapiens (human)	KEGG	7
Nitric oxide stimulates guanylate cyclase	Reactome	4
Smooth Muscle Contraction	Reactome	5
Vascular smooth muscle contraction - Homo sapiens (human)	KEGG	8

S3 Table. Summary of pathway analysis results for the shared gene list.

Pathway size	Q-value	P-value
132	0.03675	0.009769
69	0.03675	0.009486
63	0.030715	0.007387
63	0.030715	0.007387
60	0.029986	0.006453
52	0.021335	0.004321
104	0.021335	0.004247
47	0.0183	0.003243
93	0.017269	0.002842
42	0.015463	0.002349
143	0.015168	0.002112
85	0.015168	0.002047
33	0.010208	0.001163
124	0.010208	0.001124
240	0.008857	0.000785
2432	0.008857	0.000704
174	0.001807	0.000114
167	0.001747	8.85E-05
23	0.000318	1.21E-05
39	0.000173	4.38E-06
133	0.00015	1.90E-06
