# Supplementary material for: A novel method for subgroup discovery in precision medicine based on topological data analysis
Source: BMC Med Inform Decis Mak. 2025 Mar 19;25:139. doi: 10.1186/s12911-025-02852-9 (PMC11921513; doi:10.1186/s12911-025-02852-9)
Supplement: Supplementary file 5 — Supplementary Material 5: S5 Data access and pre-processing [file 12911_2025_2852_MOESM5_ESM.pdf]

A novel method for subgroup discovery in precision medicine based on topological data analysis

Supplementary File

Data accession and pre-processing

The METABRIC breast cancer cohort constituted our discovery dataset [1] (EGA, Dataset ID EGAD00010000162). Raw gene expression data (in IDAT format) were processed, quantile-normalised and log2 transformed using the beadarray R package [2]. Probe-level expression values were averaged across 18930 genes and z-scored. The METABRIC and TCGA breast cancer cohorts were restricted to include only patients that had hormone receptor positive tumours. Survival times for breast cancer patients were censored after 10 years. In total, 1,429 ER+ patients were investigated from the METABRIC dataset. For the TCGA validation dataset [3], raw TCGA breast cancer (BRCA) fastq files were downloaded from the National Cancer Institute Genomic Data Commons Data Portal legacy archive (https: //portal.gdc.cancer.gov/legacy-archive/search/f, TCGA project access number 16762). Reads were aligned to the human genome (hg38) using STAR v2.7.3a [4]. Raw counts of reads were mapped to 19,957 genes, calculated using HT-Seq (http://www-huber.embl.de/ users/anders/HTSeq/doc/overview.html). Raw counts were normalised using DESeq2 [5], log2 transformed and z-scored. For TCGA, the dataset consisted of 790 ER+ individuals. For our GTEx healthy breast tissue dataset, raw gene read counts (GTEx Analysis 2017-06- 05 v8_ RNASeQCv1.1.9 gene reads.gct.gz) were downloaded from https://gtexportal.org/ home/datasets. The dataset was restricted to include only 168 breast tissue samples and counts for 36,043 genes were normalised using "DESeq2", log2 transformed and z-scored. All gene expression datasets were converted to z-score format to minimise the discrepancy between different sequencing platforms and to standardise values [6]. Z-score measures the standard deviation σ of a data point from the population mean μ, $Z = \frac{X-\mu}{\sigma}$. Missing data was present in the TCGA and GTEx gene expression datasets. The proportion of missingness in the TCGA data was low (< 500 genes) and missing values were imputed using the KNN algorithm with $k = 10$. Genes with missing values were removed in the GTEx dataset as a large proportion of data had empty values ($> 2,500$ genes).

References

[1] Curtis C, Shah SP, Chin SF, Turashvili G, Rueda OM, Dunning MJ, et al. The genomic and transcriptomic architecture of 2,000 breast tumours reveals novel subgroups. Nature. 2012;486(7403):346–352.

[2] Dunning MJ, Smith ML, Ritchie ME, Tavar'e S. beadarray R classes and methods for Illumina bead-based data. Bioinformatics. 2007;23(16):2183–2184.

[3] Koboldt D, Fulton R, McLellan M, Schmidt H, Kalicki-Veizer J, McMichael J, et al. Comprehensive molecular portraits of human breast tumours. Nature. 2012;490(7418):61–70.

[4] Dobin A, Davis CA, Schlesinger F, Drenkow J, Zaleski C, Jha S, et al. STAR ultrafast universal RNA-seq aligner. Bioinformatics. 2013;29(1):15–21.

[5] Love MI, Huber W, Anders S. Moderated estimation of fold change and dispersion for RNA-seq data with DESeq2. Genome biology. 2014;15(12):1–21.

[6] Cheadle C, Vawter MP, Freed WJ, Becker KG. Analysis of microarray data using Z score transformation. The Journal of molecular diagnostics. 2003;5(2):73–81.
